# A Social Media Intervention for Promoting Oral Health Behaviors in Adolescents: A Non-Randomized Pilot Clinical Trial

**DOI:** 10.3390/oral3020018

**Published:** 2023-05-09

**Authors:** Susana J. Calderon, Carissa L. Comnick, Alissa Villhauer, Teresa Marshall, Jan-Ulrik Dahl, Jeffrey A. Banas, David R. Drake

**Affiliations:** 1 Mennonite College of Nursing, Illinois State University, Normal, IL 61790, USA; 2 Iowa Institute for Oral Health Research, College of Dentistry, University of Iowa, Iowa City, IA 52242, USA; 3 School of Biological Sciences, Illinois State University, Normal, IL 61790, USA

**Keywords:** oral health, adolescent, tooth brushing, text message, smartphone

## Abstract

Poor oral hygiene and excessive consumption of soda are among the main drivers of systemic health issues in adolescents in the United States. This non-randomized pilot clinical trial focused on the effects of a health text message system and smartphone-based intervention on adolescent tooth-brushing behavior and dietary choices, with a convenience sample of 94 participants aged 12 to 14 years old. A group of 75 participants agreed to use a tooth-brushing app and received a health text message; the other group of 15 agreed to use the tooth-brushing app, but did not receive a health text message. Saliva specimens were collected directly before and at the end of each experiment; changes in the salivary presence of cariogenic bacteria over the duration of the study were evaluated and compared with the demographics and behavioral variables. Within the text message group, 5% of participants increased the frequency of daily tooth brushing. Within the non-intervention group, 29% of participants increased the frequency of their daily tooth brushing. There were reductions in the total salivary bacteria and total streptococci in both groups (*p* < 0.001), but no change in the presence of cariogenic *Mutans streptococci.* Raising adolescents’ consciousness of oral health behavior resulted in marginal to moderate improvements to oral hygiene and dietary choices, as well as reductions in total salivary bacteria.

## 1. Introduction

As poor oral health is linked to systemic health issues such as cardiovascular disease and diabetes mellitus, it is important to intervene early in life to promote healthy oral health behaviors. Despite the use of fluoride and sealants [1], 57% of adolescents in the United States have caries in their permanent teeth [2]. Adolescents’ high risk for caries is associated with poor hygiene and high consumption of soda [3]. In lieu of the recommended two minutes of brushing, adolescents often apply excessive pressure for less time, leading to problems that range from gingivitis to chronic diseases [4,5]. Adolescents with acute or chronic oral disease will experience pain that impacts nutrition. Furthermore, dental caries, mouth pain, and dental visits lead to lower rates of school attendance and poorer academic performance [6].

The adolescent years are critical in terms of developing healthy habits that will have an impact as these individuals transition into adulthood. Adolescents are, therefore, an appropriate population in which to study the effects of interventions to improve oral health behavior. To effectively reduce dental caries in childhood and adolescence, we need to develop a better understanding of the factors influencing oral health behaviors. Although significant research has examined behavioral interventions to improve oral health behavior, most intervention models use conventional educational techniques and concentrate on children 2 to 7 years old. Researchers have described the lack of oral health research on adolescents and the resulting barrier to implementing oral health and wellness interventions [7]. One recent study on adolescents showed that text messaging improves compliance with tooth brushing [8]. Although teenagers spend up to 9 h a day on their smartphones and are, therefore, ideal for testing a smartphone application or “app”, there is limited research to date that has explored using social media-based interventions for adolescents to improve oral health. Smartphone apps have been tested with other age groups, and text messaging and electric toothbrushes have been validated in other studies [9,10], but no research has combined these two into one behavioral intervention.

This study seeks to shift current clinical practice paradigms by using a combined behavioral intervention of text messages and a smartphone-based app for adolescents to improve their oral health behavior and dietary choices. Using electronic toothbrushes with facial recognition and/or sensor brushes, we combined a text message system and a smartphone-based app to educate, motivate, and remind teens to brush at least twice daily and to avoid sugary drinks (by encouraging alternatives and reducing the amount and frequency). We report the results of a non-randomized pilot clinical trial, the objectives of which were to observe the effectiveness of a text message to a smartphone on increasing the frequency and duration of tooth brushing.

## 2. Materials and Methods

### 2.1. Non-Randomized Pilot Clinical Trial Design

This was a non-randomized pilot clinical trial design. Our clinical sites were two public junior high schools in the Midwest. The University of Iowa Internal Review Board reviewed and approved the study protocol (IRB#201906739), and the participants’ consent and assent was obtained. The Iowa City Community School District approved the study and provided a letter of agreement. We followed the guidance of the Consolidated Standards of Reporting Trials (CONSORT) group recommendations to warrant the rigors of the design and the reporting of the findings. Prospective participants who met the inclusion criteria were contacted; if interested, both parental/guardian consent and participant assent were obtained prior to participation in the study. Participants or legal guardian/representatives unable or unwilling to give written informed consent were excluded automatically.

### 2.2. Participants, Setting, and Recruitment

We recruited adolescents 12–14 years old, of any gender, ethnicity, race, or socioeconomic status, from the Iowa City Community School District’s two junior high schools. Our recruitment process ensured as much representation as possible among these groups by following the guidance for equitable selection of participants. We attempted to recruit potential participants who were able to answer the study’s scientific questions and represented an inclusive population; we also selected public schools that qualified for free and reduced lunches. In the US, free or reduced lunch is an indicator of the household category of lower-income families. The school nurses in each junior high school were our key informants on site, and worked with us to assist in establishing rapport with and obtaining signed consent from the participants and their parents. Access to students was offered under the oversight of the school nurse’s office. The research team was available to answer questions regarding the study via phone, text, or email. The participants were recruited with the collaboration of school nurses. Advertising consisted of flyers posted on school premises and on a virtual announcement board. We worked collegially with administrators in the school district without interfering with their daily work duties. We first attempted an intervention with a focus group (*n* = 10), and, after consulting with our gatekeepers at the school, were granted permission to carry out the intervention.

In addition to having a smartphone, the inclusion criteria for participants were: (1) aged 12 to 14 years; (2) able to provide assent, with a parent/guardian able provide consent; and (3) able to communicate in oral and written English. Exclusion criteria were use of antibiotics, oral inhalers, steroids, or non-steroidal anti-inflammatory drugs (NSAIDS) in the month preceding the study.

Prospective participants who met the inclusion and exclusion criteria were contacted; if interested, both consent and assent were obtained prior to participation in the study. Participants or legal guardians/representatives unable or unwilling to give written informed consent were excluded automatically. Participants were compensated for their participation by receiving the electric toothbrush, and they were free to withdraw from the study at any time and keep the smart toothbrush. At baseline, the participants that texted back replying with the word “Yes” were enrolled in the study. Via text message, these same participants received a complete video orientation of the technology related to the intervention.

Our recruitment goal was 100 adolescents; our eligible participants numbered 97. Three adolescents failed inclusion criteria and were ineligible. Our final total was 94 adolescents. Based on the previous literature, we performed a post hoc power analysis for the observed effect outcomes. After enrollment was completed, all covariate measures at baseline were tabulated and compared to examine the baseline comparability between the two study groups. Of the 94 subjects, 90 who met the inclusion criteria were enrolled in the study, as 4 participants later withdrew consent. Three additional participants did not participate in the final set of endpoint questionnaires/data collection, so the final total sample size was 87.

### 2.3. Group Allocation

Participants self-selected to either the control group or the experimental group. Due to our experience in studying adolescents during the recruitment phase, the research team chose participants using a self-selected sampling technique. For seven weeks, the experimental group received the intervention in the form of frequent text message reminders about dental and dietary education that text message decreased as the study progressed, while the control group received no text messages. Endpoint data were collected approximately four weeks after the last intervention. All adolescents in the study received an Oral B Genius electronic toothbrush, equipped with technology for recording the frequency and duration of tooth brushing in each oral quadrant via Bluetooth and the Oral B© app. After the orientation video, all participants also downloaded and signed in to the Oral B© app so that brushing would be automatically and electronically measured. Prior to the study, the laboratory tested the Oral B smart toothbrush for two weeks to assess its ability to collect data from users.

At both the outset of the study and its conclusion, we collected a brief questionnaire from all participants regarding their oral hygiene and dietary habits, data on their body mass index (BMI), and saliva samples to examine for their bacteria profile. During the entire study, data from the tooth brushing app were collected along with self-reported data regarding the consumption of sugary beverages.

### 2.4. Bias Reduction

The community school setting of the intervention made it challenging to conduct a non-randomized pilot clinical trial design, so the participants self-selected to either the control or the interventional group [11]. Otherwise, we followed the rigorous guidance recommended for an RCT intervention: (1) the control group did not receive the health text messaging; (2) the interventional group did receive the health text messaging; and (3) both the control and interventional groups used the Oral B Genius© automatic toothbrush and downloaded the Oral B© app for tooth brushing frequency and duration.

Due to the nature of this study, the participants selected for the intervention were not blinded. We reduced bias by not discussing details of the study while it was ongoing and not commenting on the placement of participants relative to each other. During the analysis and reporting of the collected data, we followed rigorous methods to ensure that the findings were objective.

### 2.5. Intervention and Outcomes

The intervention used an Oral B© smartphone app for tooth brushing and an SMS text message system for healthy tooth brushing and dietary choices. The system automatically sent messages such as, “Did you brush today?”, “How many times did you brush today?”, “Did you drink water today?”, and “How many sugary drinks did you have today?” The messaging was concentrated with highest frequency during the first week of the study, and gradually became less frequent over the course of the 7 weeks of the intervention.

Both the app and text messaging system were compatible with both iOS and Android. The system was implemented in Python using the Django web framework. Text messages were sent to participants using our custom-built bidirectional messaging system via a commercial web-to-SMS gateway https://www.twilio.com/en-us (accessed on 30 October 2019) [12,13]. Responses were routed back to the server in the same way. Responses were time-stamped upon receipt and automatically inserted into a database.

We looked at the intervention of the health text messages in relation to five outcomes: increase in tooth brushing frequency using the Oral B smart toothbrush, decrease in sugary beverage consumption, increase in non-sugary beverage consumption, maintenance of or reduction in body mass index (BMI), and change in bacteria.

### 2.6. Data Collection

The data collected were (1) the frequency and duration of tooth brushing on a daily and weekly basis; (2) the frequency and quantity of sugary beverage consumption, self-reported at the baseline and the endpoint; (3) saliva samples for enumeration of total cultivable bacteria and total streptococci at the baseline and the final visit; and (4) body mass index (BMI, according to weight and height) at the baseline and the endpoint. As our study has a component of assessing the consumption of sugary drinks—which is associated with caries and obesity—we added the BMI measurement in order to classify participants as obese or overweight, for example.

Demographic data (including age, gender, parents’ education level, ethnicity/race, and smoking status) were collected by self-report using a questionnaire at the beginning, when participants registered for the study. After registration and at the endpoint, participants were given a short oral health behavior questionnaire on site to collect data on the initial and endpoint tooth brushing frequency and sugary drink consumption. Both questionnaires had been used in prior studies and were validated by our expert team.

For tooth brushing, those who brushed after meals or in the morning and before bedtime were considered to have brushed twice per day, those who indicated brushing only in the morning or only at bedtime brushed once, and others were unknown.

Both the control and interventional groups sent reports of their tooth brushing frequency and duration from the toothbrush Oral B© app daily and weekly. The data from the adolescents’ daily logs and weekly tooth brushing activity was collected in a secure server, protected by the institution, to which only research team members had access.

At the baseline and the endpoint, we measured height and weight and calculated body mass index (BMI) using the CDC calculation for pediatrics, and we collected saliva samples for analysis. The two schools had different models of scales, and one school reported participants’ height verbally, so we separated the schools during BMI analysis. There were approximately 3 to 4 weeks between the last intervention and the final collection of data.

### 2.7. Biological Oral Samples

Saliva samples were collected from all study participants (*n* = 90) in 50 mL conical tubes containing 10 mL sterile buffered normal saline at both the baseline and endpoint visits (*n* = 174). Each participant was given a tube of saline and asked to swish it around in their mouth for 30 s, then to expectorate the saline back into the tube. Immediately following collection, the samples were transported to the lab for processing in a cooler containing ice packs.

The samples were vortexed for 30 s, diluted, and plated onto 3 agars using an Autoplate© Spiral Plating System (Advanced Instruments, Inc., Norwood, MA, USA). The samples were plated onto Anaerobic Blood Agar-CDC Formulation (Remel, Lenexa, KS, USA) to determine the total cultivable flora, MSKB (mitis salivarius kanamycin bacitracin) agar for enumeration of *Mutans streptococci*, and selective strep agar (Hardy Diagnostics, Santa Maria, CA, USA) to determine the total streptococci count. CDC blood agar plates were incubated anaerobically (48 h, 37 °C, anaerobic gas mix—5% CO^2^, 5% hydrogen, 90% nitrogen) in a Bactron900 anaerobic chamber (Sheldon Manufacturing, Inc, Cornelius, OR, USA), followed by 24 h of aerobic incubation (37 °C, 5% CO^2^). The selective step agar (SSA) and mitis salivarius kanamycin bacitracin agar MSKB plates were incubated for 48 and 72 h, respectively (37 °C, 5% CO^2^). Bacterial counts were determined using standard spiral plating methodology and expressed in colony-forming units per ml (cfu/mL).

### 2.8. Statistical Analysis

We first examined the baseline characteristics of the respondents to determine whether there were differences according to gender, race, grade, school, or body mass index (BMI). Baseline characteristics included brushing behaviors, dental visits, salivary bacteria levels, and types of sugary drinks consumed. Categorical variables were examined using chi-square or Fisher’s exact tests, and continuous variables were evaluated using Spearman correlation tests, Wilcoxon rank sum tests (when the independent variable was binary) or Kruskal–Wallis (when the independent variable had more than two categories). Summary statistics are reported as N (%) for categorical variables and mean (standard deviations) for continuous ones. To determine the total *Mutans streptococci* (MSKB), two separate analyses were performed: one with 0 counts included as well as one where they were excluded. We report the results for which 0 counts were excluded. Statistical analyses were performed using R version 4.0.0 and a 5% significance level.

We considered the intervention of the health text messages in relation to five outcomes: (1) increase in tooth brushing frequency using the smart Oral B toothbrush, (2) decrease in sugary beverage consumption, (3) increase in non-sugary beverage consumption, (4) maintenance of or reduction in body mass index (BMI), and (5) change in bacteria.

## 3. Results

### 3.1. Recruitment

**Figure 1.**
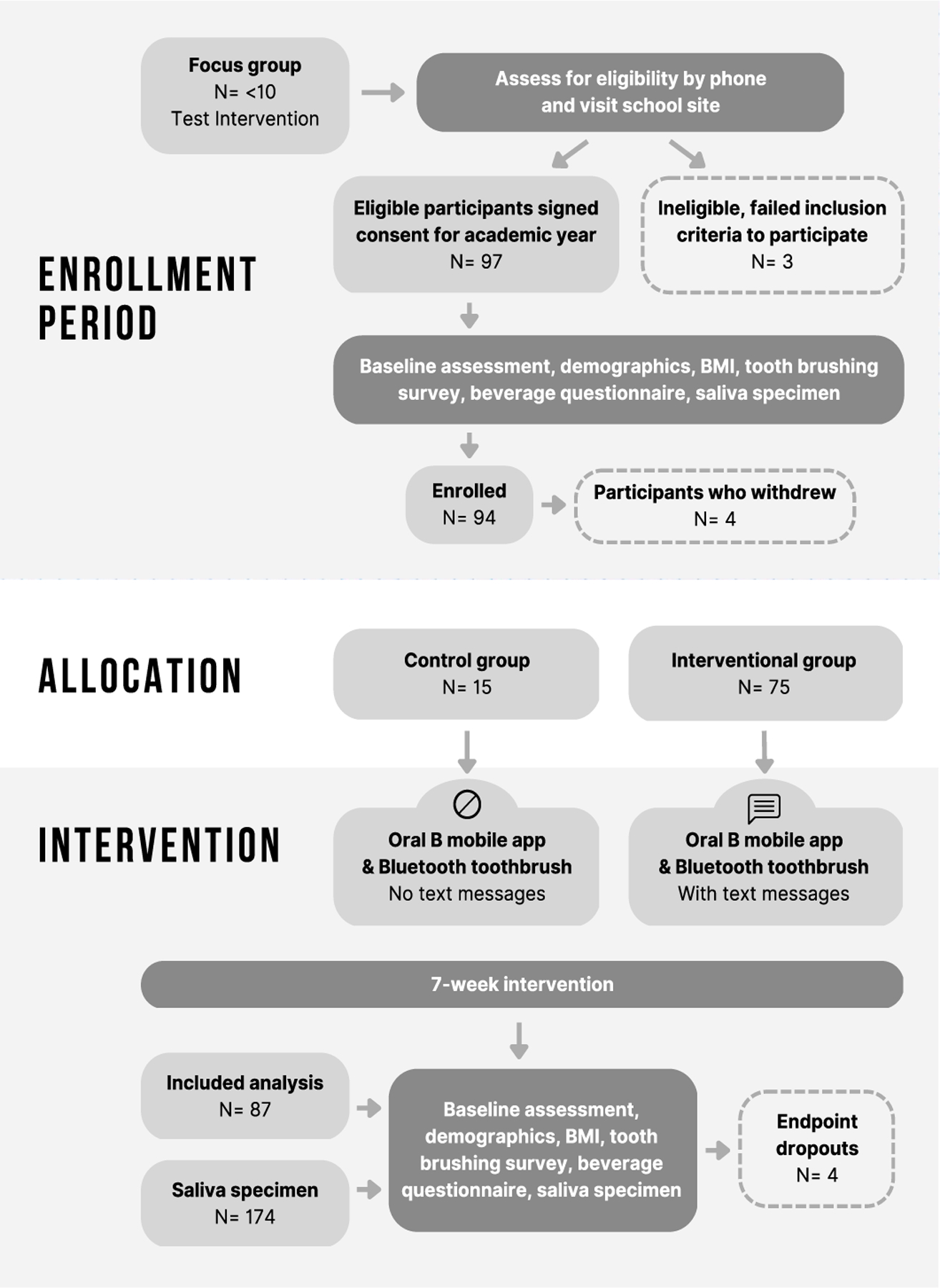
Study design flowchart—CONSORT.

In this non-randomized pilot clinical trial, a convenience sample of 94 participants aged 12 to 14 years old met the inclusion criteria to receive a health text message and use a tooth-brushing app, or to use the tooth-brushing app without the text messages, for 7 weeks (Figure 1). The allocation was 75 participants in the text message interventional group and 15 participants in the no-text message control group (4 subjects dropped out after enrollment but prior to beginning the study). The participants completed an oral health questionnaire and a sugary beverage consumption questionnaire, and saliva specimens were collected at the baseline and endpoint of the study.

### 3.2. Sample Description

There were 90 junior high school participants in this study at the baseline, of whom 87 had measurements taken at the endpoint. There were 75 (out of 90) self-selected students who opted in to receive text messages. They ranged from 12 to 14 years old. The majority (52%) were white/Caucasian (see Table 1). At baseline, 65.6% had people in their household who went to college, and 75.6% had been to the dentist within the past 6 months. The self-reported results of the demographic data (including age, gender, education level, ethnicity/race, jobs, and dentist visits) are shown in Table 1.

### 3.3. Tooth Brushing Frequency

There were no significant differences from the baseline to the endpoint between the intervention and non-intervention groups for bacterial counts, sugary or non-sugary drinks, or body mass index (see Table 2). Interestingly, there was a significant difference in tooth brushing frequency, with a higher proportion of those who did not receive the intervention reporting more frequent tooth brushing at the end of the study than at the beginning (*p* = 0.02). Of the non-intervention group, 29% reported an increase in tooth brushing frequency, while only 5% of those in the intervention group did. In the entire study, this equated to 9% of individuals improving their tooth brushing frequency.

Bacterial counts were also determined and analyzed against changes in brushing habits. There were no significant differences found.

### 3.4. Oral Bacteria Proportion

**Figure 2.**
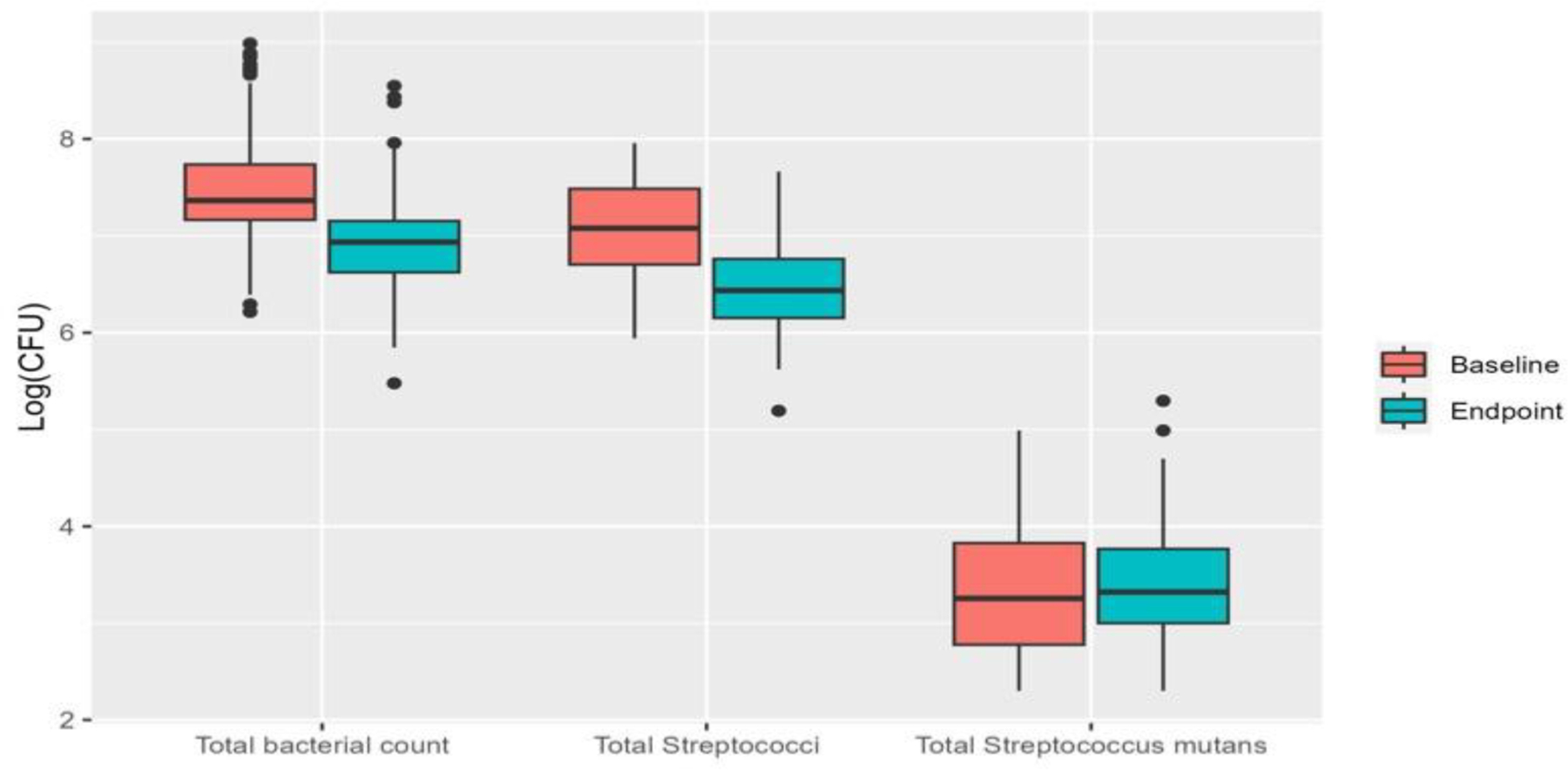
Bacterial proportion.

We examined the carriage of *Mutans streptococci*, organisms linked to a higher risk for dental decay, as well as the salivary counts of total bacteria and streptococci. At baseline, there was a significant gender difference in total *Steptococcus* strains: females had lower total bacterial counts than males (*p* = 0.005). There were no other significant differences in bacterial measures at the baseline. Over the course of the study, there were significant reductions in both total bacterial count and total streptococci (Figure 2), but not in *Mutans streptococci*. These reductions did not significantly differ between the intervention and non-intervention groups.

## 4. Discussion

The proposed study tested the efficacy of a health text messaging intervention on positively influencing oral hygiene and dietary choice behaviors. The implicit assumption is that by improving these behaviors over the short term, children will form good, long-term health-related habits, thereby decreasing their risk of future dental decay and related systemic health issues. Our results validate that in the short-term, oral health behaviors such as tooth brushing were somewhat improved among the non-interventional group of junior high students, suggesting that enrolling in the study and receiving the toothbrush alone prompted an improvement in oral health behaviors. Our assumptions were that pre-teens who brushed their teeth more would have less cariogenic bacteria and would decrease their consumption of sugary drinks. We were able to show a significant reduction in bacteria across all participants. Since the participants were drawn from public schools, many of them lacked access to dental care; were from low-income households; and did not know about positive oral health habits, including the negative effects of consuming sugary beverages. These characteristics are known to result in significant oral health disparities.

Access to preventive dental care in the form of twice-a-year visits to a dentist also varied among participants. More than 70% had two working parents and had been able to visit the dentist within the previous six months. These participants lived in an urban area in which state-funded dental services were offered. In addition, there was a higher literacy level in this area, with participants’ parents having attended college. In contrast, some participants experienced a language barrier, as the questionnaire and instructions were administered in English.

Regarding the recruitment of diverse adolescents, we found that peer group influence and one’s inner circle of friends helped to spread the word about the study. Similar findings have been reported for Hispanic recruitment, for which face-to-face and personal approaches are important emerging recruitment strategies [14]. Our study followed the US trend; study participation was mainly Caucasian, followed by African-American.

In our study, the frequency and duration of tooth brushing were obtained on a weekly basis. Participants claimed they brushed their teeth more frequently by the end of the study, and we observed that the amount of overall cariogenic bacteria was reduced. However, this change was not associated with any change in reported brushing or consumption of sugary beverages.

The study took into consideration diversity and inclusion of adolescents without judging their socioeconomical status, and extrinsic factors may have affected their participation in the interventions. Many participants were excited to receive the smart toothbrush, but several of them were found not to own a cell phone after reporting that they did own one. These participants had a pay-as-you-go phone plan or borrowed a parent’s phone, and, thus, were unwilling to receive text messages. Another study found similar findings, in which the economic level was taken into account to increase the retention of participants during the study [15]. Future studies need to consider ways to account for differences in family resources.

Similar studies have shown the difficulties of interventions that take place in a community setting [16]. Meeting adolescents in the environment in which they are comfortable is a more promising type of intervention. For the study, we assumed that adolescents who brushed their teeth would have less cariogenic bacteria and would drink fewer sugary beverages. This intervention included tooth brushing education and incorporated behavioral science insights related to sending text messages to adolescents that were intended to promote oral health, increase awareness of dental hygiene, and encourage them to replace sugary beverages in their diet. The study identified adolescent behavioral changes following the interventions.

The oral health text messages supported the education of the participants on simple changes in habits, such as switching from sugary drinks to water, and reminded them that brushing one’s teeth provides one with a healthy smile. In a study conducted in Brazil, two groups who received oral health education through educational videos versus text messaging for oral health using WhatsApp on their smartphone were compared [17]. The results of this study showed that both groups exhibited reductions in plaque indices and that the interventional group who received text messaging flossed more often than the control group after the intervention [17]. Because smartphone applications are widely used, the adolescents in this study were able to communicate more openly and have their questions answered. In our study, we noticed the participants engaging in back-and-forth texting with the research team as they reported their tooth brushing behavior, and noted that they had met their daily goals of brushing, flossing, and making sugarless drink choices. We discovered that participants who did not receive the oral health text messages brushed their teeth more frequently at the end of the study as compared to the beginning. Across all participants, the amount of observed overall cariogenic bacteria was reduced.

Certain dietary habits, such as the intake of simple carbohydrates, provide an ideal environment for dental caries [18]. Sugary beverages are high in simple carbohydrates and are easily available in stores and vending machines, increasing the consumption of these beverages and impacting the oral health behaviors of adolescents. Due to adolescents’ communication habits, targeted text messages could be one tool to decrease the consumption of sugary beverages and to improve oral health in this population.

In this study, a higher number of females—averaging 12.5 years old—participated in the study, compared with our previous study, which included more males, but a similar age group of 13 years [17]. In addition, these researchers found that text messages with an educational component yielded better results in reducing their oral plaque upon dental plaque scoring. Moreover, the use of a platform similar to text messages, such as WhatsApp Messenger, offers a user-friendly way to receive educational messages and determine the results in terms of the changes in the oral hygiene habits of participants [17]. A study found that weekly text messaging is a simple approach to increase oral health habits [8]. Some participants in this study who were in a similar age range as those in our study noted the benefits of improving their oral health habits. Moreover, the benefit was not only for oral health habits, but also improved school performance and attendance [8,17].

Interestingly, males had higher counts of total streptococci than females. Similar findings regarding gender differences showed that males had more *Mutans streptococci* for 3 and 6 months compared to females, and siblings had more *Mutans streptococci* detected due to the consumption of sugar-containing milk at 3 and 6 months [19]. There were significant differences according to gender in brushing behavior. Females had a higher rate of daily brushing, and males were more likely than females to brush only once in a while or once a day. Almost 75% of females brushed both in the morning and at bedtime, while only 47% of males did. There have been similar findings in which females increase their tooth brushing over time in comparison with males [20]. Our study showed that males consumed more sugary drinks than females did.

Participants’ body mass indices (BMIs) were measured at baseline and at the final visit. The results showed the need for cohesive measurements between sites and participants. We anticipated that the use of a similar scale and zeroing the equipment when taking anthropometric data would result in reliable information; however, missing information on body mass index (BMI) is not uncommon, as is pointed out in the study cited in [20]. Nevertheless, the participants were engaged in the process, and it opened up the opportunity to reach these adolescents and establish communication.

There was a significant gender difference in *Streptococcus* species counts in this study. Males had a higher mean count than females in the oral microbiology samples for quantification of low pH streptococci at the baseline and the final visit. It is important to note that tooth brushing frequency and consumption of sugary drinks were different among genders as well, possibly driving this difference in bacteria. This gender difference was observed in another study, in which higher levels of cariogenic bacteria occurred with worse oral habits [21]. Moreover, the role of *Mutans streptococci* in the etiology of caries is still worth considering [22].

Overall, the proportion of cariogenic bacteria decreased in this short intervention; however, there were changes in the total streptococci and total bacteria count, but not in *Mutans streptococci*. The health text message helped to remind participants to brush their teeth and to limit the number of sugary beverages consumed, but was not shown to be significantly associated with primary outcomes. The overall proportion of participants in the study who brushed twice daily increased, indicating some effect of using the smart toothbrush and smartphone app rather than receiving text messages. This oral health intervention has components of education and bringing awareness to oral hygiene and better choices regarding sugary drinks by our health text messages, which were sent on different days during the week.

Researchers showed interesting results on the lack of retention regarding the frequency of tooth brushing in both groups [17]. In the absence of intervention, the rate of compliance with a tooth brushing recommendation of 2 min twice daily is 35% to 72%. Moreover, an increase in tooth brushing twice a day in adolescents was observed in a longitudinal study [20]. Nevertheless, the finding of our study provides us with an approach to assist adolescents with their oral health habits and contribute toward decreasing the burden of oral health in adolescents.

The primary limitation of this study is its non-randomized nature. Because participants self-selected into the intervention/non-intervention groups, the results are biased by any differences between participants who chose to receive the intervention and those who did not. We cannot guarantee the two groups were similar at baseline, or whether differences in behavior over the course of the study were due to the intervention or to other factors introduced by the non-randomized nature of the trial. This limited us in obtaining meaningful data on the differences between groups by intervention status, although we were still able to look at the overall trends of the whole study population. We also did not perform sample size estimation or a pilot study, so the study may not have been powered appropriately to find differences in groups. Limitations in this study were also related to submitting the self-reported data via email. Some of the participants struggled to email us the weekly report due to the extra steps required to forward both daily and weekly reports of tooth brushing habits. This may have occurred due to a loss of motivation. In addition, we had a limited budget with which to compensate the participants. Lower socioeconomic status affected some of the participants, as not all had a cell phone with data, some used pay-per-use, and some struggled with losing the toothbrush device due to frequent moving over the weekend. Unfortunately, the interpretation of assignments at baseline was difficult, because some participants dishonestly reported owning a smartphone or were borrowing one. The time frame was constrained due to the school year, but we obtained meaningful data nevertheless. Despite all of these limitations, we were able to increase the daily tooth brushing behavior of the participants.

## 5. Conclusions

This seven-week-long non-randomized pilot clinical trial showed non-significant improvement in adolescents’ tooth brushing behavior using a smartphone-based behavioral toothbrush and app. Recruiting and retaining the youth population for studies in the US is challenging. We had significant participation in this study, showing that we are able to implement the intervention within this group if we engage them as part of the study. In our study, we obtained a very ethnically diverse population, comprising participants in two schools. The complexity of a short clinical study in the community is dependent on the dynamic of the adolescents. New studies should be performed with representative samples of students who attend public and private schools and have different socioeconomic statuses.

Nevertheless, it is important to consider the potential impact and potential role of this intervention in different environments. With regard to public health and oral health promotion, this study emphasized the potential advantages of incorporating technology into oral health interventions targeting adolescents. Although this particular intervention did not produce significant results, future research could be more effective based on this approach. From an educational point of view, the results of this study could inform the development of educational materials and programs aimed at promoting oral hygiene habits in adolescents. Furthermore, the use of technology in oral health education can motivate young people, who are often heavy users of technology. The study focuses on the importance of continuing to explore and integrate technology into oral health interventions for dentists. The use of smartphone-based tools may improve the effectiveness of dental procedures and the efforts to promote oral health, resulting in better results for patients.

Our recommendations for future directions include expanding on the research; building upon this finding; and including an interdisciplinary team with experience in nursing, microbiology, dentistry, nutrition, and biostatistics.

## Figures and Tables

**Table 1.** Sample Demographics.

Characteristics (*n* = 90)		Percentage
Gender	Male	52.2%
	Female	47.8%
Age	12 years	32.2%
	13 years	63.3%
	14 years	4.44%
Grade	Seventh	33.3%
	Eighth	65.6%
Race	Caucasian	52%
	African-American	33%
	Hispanic	5%
	Other	10%
	High school	21.1%
Household Education	College or university	65.6%
	None	11.1%
Household Jobs	Mother	11.1%
	Father	12.2%
	Both parents	74.4%
	Grandparents	1.11%
	Other	1.11%
Been to dentist	Within last 6 months	75.6%
	Within 1 year	10%
	Within 2 years	4.4%
	Within 3 years	3.3%
	No	1.1%

**Table 2.** Hypothesis analysis.

	N = 87^1,5^	Control (N = 15)^5^	Intervention (N = 72)^5^	*p*-Value^4^
Change CDC^2^	*−*0.6 (0.9)	*−*0.5 (1.2)	*−*0.6 (0.8)	0.943
Change MSKB^2^	<0.1 (0.8)	*−*0.3 (0.9)	0.1 (0.8)	0.168
Change SSA^2^	*−*0.6 (0.7)	*−*0.7 (0.7)	*−*0.6 (0.6)	0.510
Decrease in sugary drinks consumed:				1.000
No	40 (46.0%)	7 (46.7%)	33 (45.8%)	
Yes	47 (54.0%)	8 (53.3%)	39 (54.2%)	
Increase in non-sugary drinks consumed:				0.245
No	73 (83.9%)	11 (73.3%)	62 (86.1%)	
Yes	14 (16.1%)	4 (26.7%)	10 (13.9%)	
BMI ^3^ maintained or decreased:				1.000
No	39 (50.0%)	7 (53.8%)	32 (49.2%)	
Yes	39 (50.0%)	6 (46.2%)	33 (50.8%)	
Tooth brushing frequency increased:				0.016 ^*^
No	71 (91.0%)	10 (71.4%)	61 (95.3%)	
Yes	7 (9.0%)	4 (28.6%)	3 (4.7%)	

1 Control group, N = 12; interventional group, N = 72.

2 Total streptococci = SSA, total bacterial count = CDC, total *Mutans streptococci* = MSKB.

3 Body mass index = BMI.

4 *p*-values were calculated via chi-square and Fisher’s exact tests.

5 Mean (sd), count (%).

* *p* < 0.05.

## Data Availability

The study datasets and analyses are available upon reasonable request by contacting the corresponding author.
